# Crystallization around solid-like nanosized docks can explain the specificity, diversity, and stability of membrane microdomains

**DOI:** 10.3389/fpls.2014.00072

**Published:** 2014-03-05

**Authors:** Rodrigo F. M. de Almeida, Etienne Joly

**Affiliations:** ^1^Departamento de Química e Bioquímica, Faculdade de Ciências da Universidade de LisboaLisboa, Portugal; ^2^Institut de Pharmacologie et de Biologie Structurale, Centre National de la Recherche ScientifiqueToulouse, France; ^3^Institut de Pharmacologie et de Biologie Structurale, Université de Toulouse III (Paul Sabatier)Toulouse, France

**Keywords:** membrane microdomains, amorphous materials, crystal seeds, crystallization, membrane transduction, rafts

## Abstract

To date, it is widely accepted that microdomains do form in the biological membranes of all eukaryotic cells, and quite possibly also in prokaryotes. Those sub-micrometric domains play crucial roles in signaling, in intracellular transport, and even in inter-cellular communications. Despite their ubiquitous distribution, and the broad and lasting interest invested in those microdomains, their actual nature and composition, and even the physical rules that regiment their assembly still remain elusive and hotly debated. One of the most often considered models is the raft hypothesis, i.e., the partition of lipids between liquid disordered and ordered phases (Ld and Lo, respectively), the latter being enriched in sphingolipids and cholesterol. Although it is experimentally possible to obtain the formation of microdomains in synthetic membranes through Ld/Lo phase separation, there is an ever increasing amount of evidence, obtained with a wide array of experimental approaches, that a partition between domains in Ld and Lo phases cannot account for many of the observations collected in real cells. In particular, it is now commonly perceived that the plasma membrane of cells is mostly in Lo phase and recent data support the existence of gel or solid ordered domains in a whole variety of live cells under physiological conditions. Here, we present a model whereby seeds comprised of oligomerised proteins and/or lipids would serve as crystal nucleation centers for the formation of diverse gel/crystalline nanodomains. This could confer the selectivity necessary for the formation of multiple types of membrane domains, as well as the stability required to match the time frames of cellular events, such as intra- or inter-cellular transport or assembly of signaling platforms. Testing of this model will, however, require the development of new methods allowing the clear-cut discrimination between Lo and solid nanoscopic phases in live cells.

## INTRODUCTION: FROM SINGER AND NICOLSON TO THE RAFT HYPOTHESIS AND BEYOND

Biological membranes are a fundamental component of all living cells. As originally proposed by Singer and Nicolson (1972), the matrix of biological membranes is a lipid bilayer, comprised of several thousand different lipid species, with their hydrophilic heads facing the outside, and their hydrophobic moieties toward the core of the bilayer. Membranes also contain a large variety of proteins, which are less numerous than lipids, but usually amount to more than half of membranes’ dry weight.

The existence of clusters of certain proteins was already described in Singer and Nicholson’s original paper (Singer and Nicolson, 1972), including for the H2 antigens which had been monitored by Frye and Edidin (1970), in their seminal study of heterokaryons, through which they established the fluid nature of the plasma membrane (PM). The existence of membrane domains, and even of solid domains, was clearly proposed in the updated fluid mosaic model published by Nicolson (1976) just 4 years later. Although those articles were each cited many hundreds of times, for the 20 years that followed, the existence of lateral heterogeneities in biological membranes was dismissed by many scientists in the field. After the observation of sub-cellular lipid sorting by van Meer et al. (1987), a turning point was the formulation of the raft hypothesis by Simons and Ikonen (1997), which became very popular among biophysicists and cell biologists. In short, this hypothesis proposed a view of membrane organization based on the existence of “rafts” enriched in sphingolipids and cholesterol, which would ensure the specific transport of particular proteins between cellular compartments, and direct the assembly of signaling platforms. One of the tenets of the raft hypothesis was that rafts were resistant to solubilization at 4°C with a non-ionic detergent such as Triton X-100. Although the approach of isolating detergent resistant membranes (DRMs) had the undisputable advantage of being based on experimental observations, this convolution of the theoretical notion of rafts with the operational definition of DRMs has generated numerous misconceptions and over-interpretations that have already been summarized by others for plants and fungi (Malinsky et al., 2013) and for animal cells (Quinn, 2010).

Before the formulation of the raft hypothesis, the idea that microdomains exist in biological membranes and may be related to the different lipid phases found in model membrane systems had already been discussed for many years (Stier and Sackmann, 1973; Nicolson, 1976; Karnovsky et al., 1982; Welti and Glaser, 1994). In initial studies, mostly based on the photophysical properties of fluorescent probes, some domains were labeled as “solid/fluid,” but it is now known that these putative solid domains would be better described as liquid ordered (Lo; Bastos et al., 2012). This Lo state is formed due to the ability of membrane-active sterols such as cholesterol to provoke an intermediate state of lipid bilayers, which are still completely fluid, but are stiffer, thicker, and less permeable to water than when in liquid disordered phase (Ld), i.e., completely fluid (Dufourc, 2008). Lipid bilayers containing sterols can still harbor gel phase domains, which will be described below, albeit at lower temperatures and lower sterol concentrations, and the term solid ordered (So) is then often used to describe them.

Despite the intense interest in studying membrane micro-domains, their precise nature – and even the physical rules (in other words the thermodynamic principles) that govern their assembly – still remains elusive and hotly debated. To date, the most commonly held view is that the formation of rafts corresponds to a situation of coexistence of Lo and Ld phase domains (see London, 2005 for review and historical account). Whilst it is possible to observe the coexistence of Ld and Lo phases in many membrane model systems, the relevance of such domains to the microdomains in membranes of live cells is still highly disputed (Elson et al., 2010). For example, in synthetic membranes where Ld and Lo domains coexist, most proteins – even those considered as raft-specific proteins – partition preferentially into the Ld phase (Dietrich et al., 2001b; Bacia et al., 2004; Kahya et al., 2005; Nikolaus et al., 2010). This is also true of many other molecules such as fluorescent probes (de Almeida et al., 2009) and drugs (Custodio et al., 1991).

The real-life structure of membranes is undoubtedly much more complex than a simple dichotomy between disordered and ordered domains. Specifically, the simplistic Ld/Lo partition model cannot provide a satisfactory explanation (i) for the formation of multiple types of microdomains within the same cell, which may even often occur simultaneously; (ii) for the recent evidence that most of the PM of eukaryotes is in the Lo phase, with cholesterol acting more as fluidifier and as a homogenizer rather than as a promoter of domains; (iii) for the fact that membrane microdomains are present in bacteria that lack both sphingolipids and sterols; (iv) for the occurrence of domains in solid or gel phase in eukaryotes under physiological conditions.

In the following pages, we will address these issues successively, and propose an alternative mechanism for the formation of highly selective and/or stable microdomains by a process of crystalline recruitment into membrane “docks” seeded by specific proteins/lipids. Membrane docks in a solid-like state may be an important seed for the formation of highly specific and/or stable membrane microdomains, but the detection of such nano-structures is a technological challenge and, maybe more importantly, will require that scientists accept the possibility of their existence.

## THE DIVERSITY OF MICRODOMAINS

The PMs of eukaryotes contain several types of microdomains; examples include not only the “standard” rafts, but also caveolae (Parton and Simons, 2007), the recently described “heavy” rafts that may be involved in T cell signaling (Hrdinka et al., 2012), cytoskeleton-dependent sphingolipid domains (Kraft, 2013), ceramide-rich platforms (Stancevic and Kolesnick, 2010), and tetraspanin-enriched domains (Yanez-Mo et al., 2009).

What follows is by no means an exhaustive review of all the different types of microdomains, but a collection of a few selected examples that either demonstrate, or are particularly suggestive of the simultaneous occurrence of different domains in the membranes of eukaryotic cells that cannot possibly be explained by Ld/Lo phase separation alone. Other examples will refer to domains that do not fit the standard definition of a raft, i.e., a microdomain enriched in sterols and sphingolipids.

In T cells, different gangliosides are needed for activation of various subsets of T cell: GM3 for CD4+ T cell activation and GM1 for CD8+ T cells (Nagafuku et al., 2012). These authors attributed this finding to the existence of different types of functional raft domains containing specific ganglioside species in each T cell subset.

In HEK cells, the simultaneous existence of multiple types of membrane domains was recently suggested by the distinct localizations of two raft-associated proteins in different regions of the PM, as revealed by total internal reflection microscopy (Asanov et al., 2010).

Whereas “rafts” are defined as cholesterol and sphingolipid-enriched domains, sphingolipid domains are not necessarily enriched in cholesterol: fluorescence lifetime imaging microscopy revealed cholesterol-independent, sphingolipid-enriched microdomains containing signaling molecules in the same membranes as cholesterol-dependent microdomains (Hofman et al., 2008). More recently, compelling evidence for the existence of cholesterol-independent sphingolipid domains has come from chemical mapping studies using isotope-labeled sphingolipids and cholesterol in fibroblasts. Whereas cholesterol was homogeneously distributed, this approach revealed the presence of sphingolipid microdomains, which disappeared after cytoskeleton disruption (Frisz et al., 2013a,b). Further evidence for sphingolipid domains is summarized in a recent review (Kraft, 2013).

The membrane microdomains in plants and fungi have features that differentiate them from what the mammalian research community calls rafts (Malinsky et al., 2013), possibly related to the fact that plants and fungi have cell walls. These domains are in general more stable than those of mammalian cells - they can last for the duration of a cell cycle, and since they are usually observed by conventional fluorescence microscopy, they are also much larger than the few nanometers usually attributed to lipid rafts. A systematic study of the yeast *Saccharomyces cerevisiae* has revealed that the PM is organized into patches and networks of numerous domains containing specific subsets of proteins with similar transmembrane domains (Spira et al., 2012). This organization of yeast membrane compartments/membrane microdomains correlates with their lipid composition and is actively maintained by the cell through energy-consuming processes (Spira et al., 2012; Malinsky et al., 2013).

Although lipid phases most likely contribute to the formation of domains, these various examples illustrate that the lateral organization of biological membranes cannot rely simply on a dichotomy between Ld phases, enriched in glycerophospholipids with unsaturated lipid chains, and Lo phases, enriched in sphingolipids and sterols. If Ld and Lo phases do not suffice to explain how lipids organize the variety of membrane domains, what does? Proteins undoubtedly play a central role in orchestrating the organization of biological membranes but, given the pivotal role of sterols in influencing the phase behavior of lipid bilayers, the following section will first explore how the biophysical properties of sterols might contribute to organizing membrane domains.

## THE ROLE OF STEROLS

Sterols bring order to the fluid phase of biological membranes, which is linked to their capacity to increase membrane impermeability to water and small ions, as well as their rigidity and solidity (Haines, 2001; Hauss et al., 2002). Yet sterols can also act as solvents and homogenizers: when above a certain threshold amount, they prevent the appearance of solid domains in a large variety of lipid mixtures and facilitate the interactions between lipids with very disparate melting temperatures (*T*_m_; some below 0°C and others as high as 60°C), with some paradigmatic examples given below.

**FIGURE 1:**
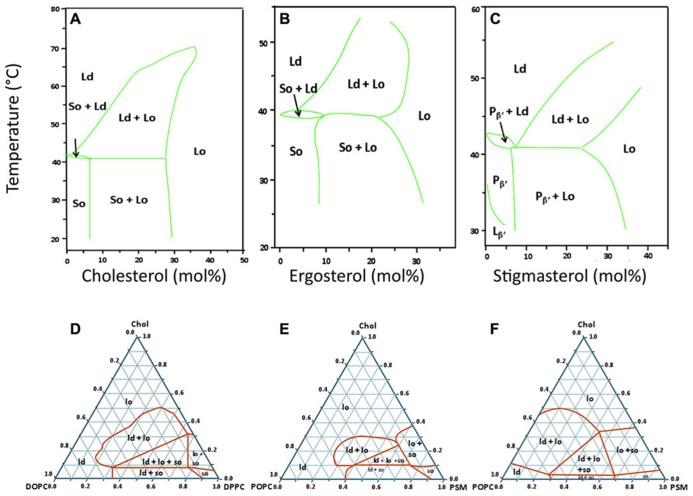
**Typical lipid phase diagrams used in the study of sterol-related membrane domains.** Top row: temperature – composition binary phase diagrams for mixtures of DPPC with three different sterols **(A)** Cholesterol (animals; Sankaram and Thompson, 1991) and references therein, **(B)** Ergosterol (fungi; Hsueh et al., 2005), **(C)** Stigmasterol (plants; Wu et al., 2006). These three diagrams were selected to show that, for the same saturated lipid (DPPC), the type of phase diagram and the regions of coexistence of Ld, Lo, and/or So phases are similar for the three eukaryotic kingdoms. For panel **C**, the techniques used allowed to distinguish two types of gel phases below the *T*_m_: the tilted gel phase (*L*_β__^′^_) and the periodic quasi-lamellar gel phase (*P*_β__^′^_). Bottom row: ternary phase diagrams for mixtures of cholesterol with two other lipids, one with a high *T*_m_ and the other with a low *T*_m_. **(D)** Typical phase diagram for the DOPC/DPPC/cholesterol system at 24°C. This mixture is one of the best characterized systems, and the results obtained by different laboratories, even when using different techniques for phase characterization are usually in very close agreement (Veatch et al., 2004; de Almeida et al., 2007). Incidentally, it was with this mixture that the coexistence of the three phases (Ld, Lo, So) in one single GUV was experimentally demonstrated for the first time (**Figure 2A**). Panels **E** and **F** are an example of divergent phase diagrams reported by two different laboratories for a similar mixture of *N*-palmitoylsphingomyelin (PSM), 1-palmitoyl-2-oleoyl-*sn*-glycero-3-phosphocholine (POPC), and cholesterol, at the same temperature of 23°C (**E**: Ionova et al., 2012; **F**: de Almeida et al., 2003). These two phase diagrams illustrate that there is no complete agreement for this mixture when different methods and techniques are used to detect regions of phase coexistence. This system is, however, considered to be more biologically relevant than the one on panel **D** since POPC, the low *T*_m_ phospholipid, has a saturated and an unsaturated acyl chain, and sphingomyelin is the most abundant sphingolipid in the PM of mammalian cells.

When most hydrated phospholipids and sphingolipids are heated, they usually undergo several phase transitions, the most important of which is from a solid-like state (also called gel) to a liquid-like state (the fluid phase). Methods such as differential scanning calorimetry can be used to determine the temperature of this gel–fluid phase transition of the bilayer, which is also called the main transition temperature *T*_m_, or melting temperature. The main transition owes this designation to the fact that it is the most cooperative and the one with the highest associated enthalpy change for phospholipids. Although different sterols populate the PM of mammalian, fungal, and plant cells, when present in a sufficiently high molar fraction [around 30 mol% in the case of 1,2-dipalmitoyl-*sn*-glycero-3-phosphocholine (DPPC)], they all have the ability to prevent the abrupt gel–fluid transition by provoking the formation of a single Lo phase (**Figures 1A–C**). Conversely, at lower concentrations, those sterols promote the coexistence of So/Lo phases below the temperature of three phase coexistence (which is slightly below *T*_m_), and the coexistence of Lo/Ld above this temperature. Thus, any lipid with a sufficiently high *T*_m_ value can, in principle and under certain conditions, form gel domains in the context of a biological membranes [e.g., phospholipids with unusually long and saturated acyl chains, and many (glyco)sphingolipids]. Moreover, it has been established that sphingolipids and glycerophospholipids can form stoichiometric complexes with crystalline characteristics and very high thermal stability (Quinn, 2010).

Pioneering studies of domain imaging in eukaryotic biological membranes suggested that these micro-heterogeneities should be in a fluid state (Rodgers and Glaser, 1991), and to date, the idea that domains in gel/solid/crystalline state could play physiological roles in biological membranes is rejected by most membrane biophysicists, but we contend that these views are mostly based on indirect arguments rather than on direct and solid grounds. First, it is widely perceived that “frozen” structures are not compatible with life but, as we will describe in later sections, nanoscopic structures in a gel/solid/crystalline state can remain extremely dynamic, especially over the timescales of cellular events, i.e., seconds or minutes. Second, many proteins reconstituted into liposomes with no sterols lost their activity when the bilayer was in the gel phase, i.e., when the temperature was below the *T*_m_ of the lipids (Welti et al., 1981). Such observations do not, however, rule out that solid domains could exist in live cells, and could even be involved in turning off the activity of certain proteins. Third, in isolated membranes or in liposomes prepared with lipids extracted from PMs of cells from various sources, the measured values of *T*_m_ seemed to be, in most cases, just below the growing temperature of the original cells or the physiological temperature (e.g., 37°C for human erythrocytes; Mouritsen, 1987). Such membranes do not, however, contain all the proteins that could play critical roles in regulating the state of the lipids. In fact, as mentioned below, there are many situations where large fractions of certain PM proteins are immobile in the timescale of fluorescence recovery after photobleaching (FRAP) experiments, and this stable location is important for their biological function. All together, those arguments are thus quite far from providing a definite proof that solid domains could not exist in biological membranes. However, since Singer and Nicholson’s fluid mosaic model suggested that the membranes of living organisms under physiological conditions should all be in a fluid state, the discovery of the Lo/Ld immiscibility provided the grounds to assume that microdomains in biomembranes would correspond to this type of phase separation (London, 2005).

**FIGURE 2:**
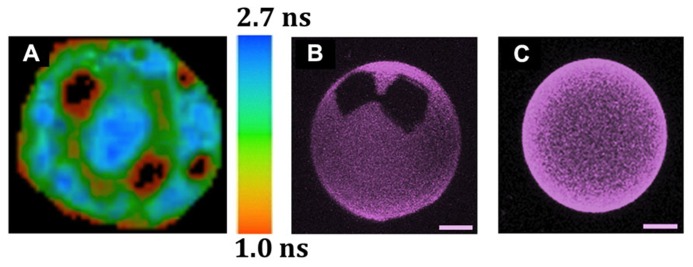
**Observation by different fluorescence microscopic techniques of lipid domain organization in GUVs and of the solubilizing properties of cholesterol.** The GUVs were all labeled with Rho-DOPE (0.2 mol%), which partitions preferentially into Ld over Lo phases, and is excluded from So domains. **(A)** Coexistence of three lipid phases (Ld/Lo/So) in one single GUV, as detected by FLIM after two-photon excitation (de Almeida et al., 2007). The image corresponds to the top view of a ternary GUV DOPC/DPPC/cholesterol 11:11:3 mol:mol:mol at 24°C. The 3 phases were identified by combining the data from fluorescence intensity and lifetimes. So (black areas), Lo: intermediate intensity and mean lifetime of ~1.7 ns (green areas), Ld: higher intensity and mean lifetime of ~2.5 ns (blue areas). The sidebar provides the fluorescence lifetime color code. **(B,C)** The presence of 12% of *N*-palmitoylceramide (PCer) results in the formation of gels domains, even with 10% cholesterol, but such domains are not seen at 40% cholesterol, even when the amount of PCer is raised to 20 mol% (Castro et al., 2009). The precise GUV compositions are **(B)** POPC/cholesterol (9:1) with 12 mol% PCer and **(C)** POPC/cholesterol (3:2) with 20 mol% PCer. The images are 3D projections obtained from confocal sections of GUVs at 22°C (scale bar: 5 μm).

The direct observation by imaging techniques of coexisting Ld and Lo phases in giant unilamellar vesicles (GUV) generated using lipids that were either synthetic or extracted from mammalian cells (Dietrich et al., 2001a) provided an important experimental support for the lipid raft hypothesis. When the cholesterol content is low (i.e., below 10%), however, all such systems making use of lipids with very different *T*_m_ values display typical gel/fluid phase coexistence similar to those found in binary lipid mixtures (**Figures 1D–F**). Although there is not always a complete agreement regarding some of the ternary phase diagrams presented by different authors (e.g., **Figures 1E,F**), it is clear that, under the right conditions, ternary mixtures comprised of the right proportions of sterol and of lipids with high *T*_m_ and low *T*_m_ can harbor co-existing Ld/Lo regions, both as planar bilayers or as GUVs. In some instances, the co-existence of gel and Lo regions is also possible, and, more recently, the possibility of having So/Lo/Ld coexistence, even in one single GUV, has been demonstrated (**Figure 2A**; de Almeida et al., 2007).

In membranes reconstituted with lipids extracted from cells, however, one should not forget that:

(i) proteins are not present in reconstituted membranes, whereas they comprise a very significant proportion of biological membranes (often more than 50% by weight), and proteins can have organizing or rigidifying properties (Harrington et al., 2012), or the capacity to act as seeds for a liquid-solid transition (Schram and Thompson, 1997; Joly, 2004; Schram and Hall, 2004). Conversely, some proteins are also known to limit the packing ability of lipids (Aresta-Branco et al., 2011) and to decrease their average transition temperature and widen the transition (Cameron et al., 1983).
(ii) the lipid composition of reconstituted membranes may not always faithfully reflect that of the original PM they were extracted from because of possible contamination with membranes from intracellular compartments or because some of the PM lipids with very high *T*_m_ may not incorporate into the reconstituted membrane as efficiently as the more fluid ones (Ali et al., 2006; Silva et al., 2006).

Sterol contents in yeast, plants and animal PM are classically above 30 mol% of lipids (van Meer et al., 2008), which actually suggests rather strongly that most of those membranes should be in Lo phase. In agreement, several groups have indeed advocated that the PM of different types of cells is probably mostly comprised of Lo phase. For example, Owen et al. (2012) have estimated that, in a T cell line, over 75% of the PM is in Lo phase. This is not to say that the PM is necessarily completely homogenous since theoretical simulations have suggested that sterol-rich Lo phases can still display nanoscopic heterogeneities (Miao et al., 2002). Another example lies with the comparison of membrane thicknesses derived from neutron scattering, which revealed small differences between isolated putative rafts and the average thickness of the membrane (4.64 vs. 4.53 nm, respectively; Quinn, 2010). This can be taken as an indication that most of the PM is in fact in a Lo-like state, and this result somehow weakens the idea that hydrophobic mismatch between lipid bilayers and transmembrane proteins could be solely responsible for sorting proteins into different membrane domains.

Perhaps the most important message that can be taken from those various studies and phase diagrams is that, above certain sterol concentrations, all lipid bilayers will tend to be in a Lo phase over broad ranges of temperatures (**Figure 1**), including membranes containing ceramides with very high *T*_m_ (Castro et al., 2009; **Figures 2B,C**). Another very important conclusion to be drawn from all these studies is that even relatively simple mixtures display complex phase behaviors. Since the PM of an eukaryotic cell is comprised of several hundred or thousand different lipid species and many different proteins, one can thus expect that they have very complex phase behaviors. Because the membranes of bacteria contain fewer lipids and no sterols, it is tempting to turn to those as an alternative model of phase behavior in biological membranes.

## MICRODOMAINS IN BACTERIAL MEMBRANES

Although bacterial membranes do not contain sterols, microdomains can clearly form in the membranes of bacteria (Lopez and Kolter, 2010). For example, functional microdomains involving a flotillin homologue have been identified in the membranes of bacteria, namely *Bacillus subtilis* and possibly also in *Staphylococcus aureus* and *Escherichia coli* (Lopez and Kolter, 2010). Given that bacteria are devoid of sterols (with a few notable exceptions mentioned below), and most of them also of sphingolipids, those domains must thus clearly differ from eukaryotic rafts. In fact, bacterial membranes seem to rely more on a solid/liquid dichotomy than on an Lo/Ld partition. Multiple reports, based on a variety of approaches, have indeed documented that, although the membranes of exponentially growing bacteria are usually in a fluid phase, solid phases tend to appear as soon as the temperature is reduced, or under a variety of other conditions. The existence of gel phases in bacterial lipid membranes was first described as early as 1975, using nuclear magnetic resonance (NMR) spectroscopy to investigate the physical state of deuterated palmitic acid which had been incorporated into the membranes of *Acholeplasma* *laidlawii* bacteria. This revealed that, just underneath the growth temperature, a significant portion of the bacterial membrane turned to a gel state (Stockton et al., 1975). Interesting studies were later performed on the same bacterial species where the ratio of lipid saturation in the bacterial membranes was increased by feeding C14:0 saturated myristic acid to either fatty acid-auxotroph bacterial strains or to WT strains in the presence of drugs inhibiting fatty acid synthesis. It was found that, even at the normal growth temperature, ~85–90% of the membrane lipids were in gel state, and that proteins had little effect on lipid order (Jarrell et al., 1982). Another study based on FTIR (Fourier-transform infrared spectroscopy), also using live *A.* *laidlawii* bacteria, showed that, at 30°C (i.e., the growth temperature), a significant percentage of lipids was in the gel phase, and that below 20°C, the membranes were entirely in gel phase, whilst very high viability was still preserved (98–99%; Cameron et al., 1983). A recent study also based on FTIR has shown that, in *Geobacter sulfurreducens, *gel phases are caused by osmotic stress or desiccation (Ragoonanan et al., 2008). Although the above studies, which are mostly based on “forced-feeding” saturated fatty acids are not entirely physiological, they do show that bacterial cells can survive and even grow containing high amounts of gel phase in their PM. In support of this, it has been estimated that *E. coli* can grow normally with as much as 20% of their membrane lipids in a gel state (Jackson and Cronan, 1978).

Although prokaryotes do not contain sterols, most of them possess various forms of branched or cyclic polyterpenoid lipids that strengthen their membranes and contribute to making them more resistant to potential toxic molecules such as alcohol, and more impermeable to water than bilayers made simply of linear lipids such as phospholipids (Ourisson et al., 1987). Among those surrogate molecules, the group of molecules known as hopanoids share many structural features with sterols. Recently, a biophysical study has shown that diplopterol, the simplest bacterial hopanoid, can interact with N-stearoyl-sphingomyelin and induce the formation of an Lo-like phase (Saenz et al., 2012).

To date, only a handful of eubacteria have been identified that have the ability to synthesize sterols, and many of those probably acquired the genes by horizontal gene transfer from eukaryotes (Hannich et al., 2011). Among those bacteria that can synthesize sterols, however, a particularly interesting phylum is that of the Planctomycetes, which carry genes for sterol synthesis that have been proposed as the likely precursors of those found in eukaryotes (Pearson et al., 2003). For a variety of reasons, Planctomycetes are increasingly perceived as the phylum that most likely gave rise to eukaryotes, presumably via an event of symbiotic fusion with an archae bacterium. For example, eukaryote-like features of the planctomycete species *Gemmata obscuriglobus* include: a large volume (3 to 5 times larger than typical bacteria such as *E. coli*); a genome of ~8000 genes, including some for ancient tubulins for a rudimentary cytoskeleton; the lack of peptidoglycan in the cell wall; and finally the presence of endomembranes, which allow those bacteria to carry out an endocytic-like process (Devos, 2013). To date, among prokaryotes, Plantomycetes are the ones with the most developed endomembrane system. There is therefore a striking correlation between the acquisition of sterols and the capacity to harbor at least two different types of membranes, one for the outer PM and one inside the cell. Of note, in eukaryotes, whilst the PM is rich in sterol, the inner membranes, including those of the nucleus and of mitochondria, are rather akin to standard prokaryotic membranes (van Meer et al., 2008; Lippincott-Schwartz and Phair, 2010) because they harbor almost no cholesterol and must thus be much less prone to harboring Lo phases. They will, however, remain in a fluid Ld state most of the time because they contain only minute amounts of sphingolipids and are mostly comprised of glycerophospholipids with low *T*_m_ such as PC and PE, with high proportions of unsaturated fatty acids (Fridriksson et al., 1999; van Meer et al., 2008).

Sphingolipids are much more widely distributed than sterols among prokaryotes, and meta-consensus analyses have revealed that the enzymes for sphingolipid metabolism are among the most widespread, and can be found in all three domains of life (Goldman et al., 2012). So far, however, there are no known prokaryotes that simultaneously harbor both sphingolipids and sterols (Hannich et al., 2011). Conversely, and rather strikingly, the PM of all known eukaryotes contains high amounts of both sterols (30 to 50%) and sphingolipids (ca. 15%; van Meer et al., 2008). Some eukaryotes, such as drosophilae, cannot synthesize sterols, but still need them and have to obtain them from their diet.

## COEXISTING SPHINGOLIPIDS AND STEROLS: FROM PROKARYOTES TO EUKARYOTES

One broadly favored scenario for the appearance of the first eukaryote is one of a symbiotic fusion event between an archae and a eubacterium, subsequently followed by engulfment of a purple bacterium that would evolve into mitochondria (Margulis, 1996). Although it was initially suggested that the heterochiral membranes that must have resulted from the fusion of a eubacterium and an archae would be very unstable, this inherent instability has not been confirmed (Lombard et al., 2012). Worthy of note, in eukaryotes, sphingolipids are predominantly found in the extracellular leaflet (Zachowski, 1993) whereas sterols, at least in some cells, are predominantly in the cytoplasmic leaflet (Mondal et al., 2009). A particularly interesting idea put forward in this latter paper is that an important role of sterols could be related to their capacity to flip very rapidly between the two leaflets, allowing them to fill any gaps that could result from metabolism or transport events, thus resulting in a better stability of the membrane.

From a lipid-centric perspective, given the information provided in the previous paragraphs, one can be tempted to suggest that a critical event in the making of eukaryotes lied with the fusion of a sterol-containing planctomycete-like eubacterium on the one hand, and a sphingolipid-containing bacterium on the other (this latter corresponding either to the presumed ancestral archae, or the mitochondrion ancestor, or another yet unrecognized fusion partner). The fusion of two cells is an abrupt event that does not allow for the progressive evolution of genes and metabolism, and the hybrid resulting from such a fusion would thus have suddenly harbored sterols and sphingolipids within a single membrane system. As we have seen above, the formation of membrane domains in bacteria involves transitions between solid and fluid states, and there is no reason to suspect that this would have been any different in the bacterial ancestor(s) which did not contain sterols, and whose fusion to a sterol-containing one gave rise to the first eukaryotes. Some of the membrane proteins from this bacterial ancestor would thus have been adapted to function specifically when membranes were in a solid state. One of the advantages of bringing sterols and sphingolipids together in the same membranes must have been that the membranes became less susceptible to all-or-nothing switches between solid and fluid phases, in particular as a consequence of variations in temperature. If the co-habitation of sterols and sphingolipids had resulted in a complete inhibition of the formation of solid domains, however, many proteins that were previously activated by the formation of a solid microdomain in the bacterial membrane would have suddenly found themselves incapable of carrying out their functions, and it is hard to imagine that the cells resulting from the fusion would have survived such a sudden functional loss. Thus, if the solid domains were present in the bacterial ancestors, they almost certainly continued to form in the earliest eukaryotic ancestors. We perceive that what the co-existence of sterols and sphingolipids provoked in the earliest eukaryotes was not only a better resistance to sudden changes in temperature, but even more importantly, that it allowed early eukaryotes to depart from dichotomic responses to embrace the possibility of forming a larger diversity of membrane microdomains. In turn, this would open the doors to the formation of different types of domains, which could be linked to different intracellular responses, and pave the way to more elaborate developmental programs indispensable for the formation of multi-cellular organisms. A major difference between plants, fungi and animals is in the nature of their sterols, which may be related to the formation of different types of microdomains, leading to very diverse cellular processes, including the different mobility of their respective cells.

In the following section, we will present the existing evidence that such crystalline microdomains (in other words, gel or solid microdomains) very probably form in the membranes of the most evolved eukaryotes of today.

## EVIDENCE FOR THE EXISTENCE OF SOLID DOMAINS IN EUKARYOTIC MEMBRANES

Since the discovery of Lo phases, gel or solid ordered domains have been almost universally considered as non-physiological and incompatible with a functional membrane in eukaryotes. To date, however, an increasing amount of evidence supports the existence of such domains in a whole variety of live cells, under physiological conditions, even if they continue to be referred to as exceptional situations (Jouhet, 2013).

In line with a hypothesis formulated by one of us some 10 years ago (Joly, 2004), we perceive that, among the whole diversity of different types of membrane microdomains, many may actually form through a process of crystallization around docks that would be mostly seeded by proteins. For example, it was shown at least in two cases that localized membrane rigidification can represent important defense mechanisms either as an initial signal following a temperature decrease in plants and other poikilotherms (Vaultier et al., 2006), or as a defense against antimicrobial peptides in yeast (Veerman et al., 2007). Whilst a membrane completely in Lo phase would be relatively insensitive to such signals, in a membrane harboring the cohabitation of Lo and Ld domains, the latter would turn into Lo domains (and/or become more rigid). This type of effect, however, would have a strong tendency to extend to the PM of the entire cell and therefore be too drastic. The formation of small seeds of solid domains, still surrounded by membranes in Lo phase, would seem a more easily tunable mechanism for providing detectable yet controllable signals.

**FIGURE 3:**
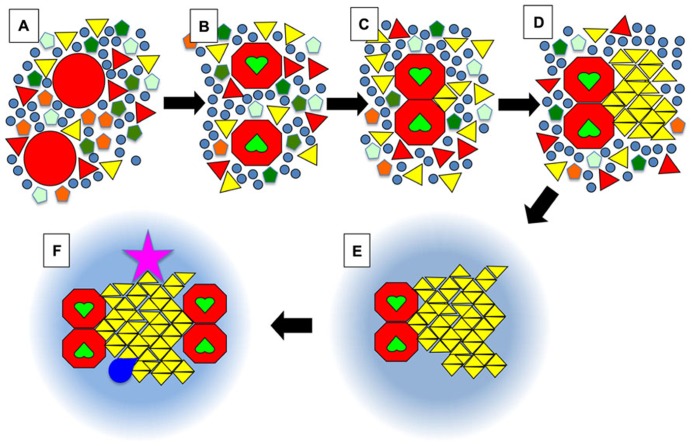
**Schematic view of the dock model. (A)** Membrane proteins in a resting state (large red circles) are surrounded by a whole diversity of lipids such as phospholipids (pentagons), (glyco)sphingolipids (triangles), and sterols (circles). Some of these lipids would have a tendency to form solid phases at physiological temperatures but in the absence of stimuli the solid nanodomains do not reach a critical size that leads to further growth into a crystalline-like structure. The symbols can also be viewed as complexes formed by a small number of lipid molecules. **(B)** Upon activation, for example when receptors bind their ligand (green heart shapes), certain proteins will undergo a conformational change. **(C)** This will favor their homo- or hetero-dimerisation. The juxtaposition of proteins will create a new environment that will promote the binding of certain lipids such as one particular type of sphingolipid (yellow triangles). **(D)** Those bound lipids will act as the seed for the crystalline growth of a membrane dock, with the crystalline mesh dictating the specificity for one (or quite probably several) type(s) of lipid(s) with particular head groups and acyl chains, e.g., certain gangliosides (in the outer leaflet), phosphatidilinositol phosphates (PIPs) or even phosphatidic acid (PA) (in the inner leaflet). **(E)** After a crystalline dock forms, it will be surrounded by a ring of lipids with intermediate properties between So and Lo, which will most likely be quite similar to what is usually defined as rafts, i.e., enriched in sphingolipids and cholesterol. **(F)** Via percolation and enlargement of the catchment area, the rings could favor the exclusion or recruitment of other activated receptors, or of cofactors (kinases, GTPases …; dark blue shape). Importantly, some components of the dock (pink star) might be anchored to the cytoskeleton on the cytosolic side, or on the exoplasmic side to the extra-cellular matrix in mammalian organisms, or to the cell wall in plants and fungi.

In yeast, FRAP experiments have demonstrated that there are PM proteins that diffuse very slowly, at speeds more compatible with gel than fluid domains (Valdez-Taubas and Pelham, 2003; Ganguly et al., 2009). In addition, single particle tracking techniques have demonstrated a complex confinement network and restricted diffusion of both lipids and proteins in animal cells. A commonly offered explanation for such behaviors is that certain proteins, linked to the cytoskeleton, are organized as corrals and pickets fences, and can hinder the diffusion of individual molecules (Kusumi et al., 2012). The architecture of such barriers, however, still remains to be fully elucidated, as well as the actual mechanism whereby proteins interacting with the cytoskeleton, i.e., in the aqueous phase of the cytoplasm, could hinder the diffusion of so many proteins and lipids diffusing in the plan of the membrane. As an alternative, we propose that some of the obstacles limiting the molecules’ diffusion could correspond to areas in a gel state (**Figures 3E,F**). Accordingly, when molecules do get transiently trapped into gel nanodomains, it is the diffusion of those nanodomains which will be limited, quite possibly by other adjacent domains, or when the solid nanodomain is anchored to the cytoskeleton by one, or just a few, of its components. Such a mechanism would suffice to explain why the cytoskeleton has been found to play a pivotal role in the hindered diffusion of proteins and lipids in a multitude of different experimental systems (Kusumi et al., 2012), and does not call for the membrane to lie on a skeleton of actin filaments in such an intimate fashion that it can limit the diffusion of many of the membrane components, including certain lipids such as sphingolipids that are mostly found on the extra-cellular leaflet. It is worthy of note that the idea that the existence of solid domains could be limiting the diffusion of membrane components had already been clearly put forward by Nicolson almost 40 years ago (Nicolson, 1976). Moreover, in the model proposed by Kusumi et al. (2012), the existence of micrometer sized corrals defined by cytoskeleton and transmembrane proteins does not preclude the existence of nanometer sized lipid domains within each of those corrals. Of note, it has been proposed that in the case of cell-walled organisms, such as plants and yeast, the cell-wall plays a role similar to the cytoskeleton in hindering diffusion of PM components, as reviewed in another paper of this issue (Martiniere and Runions, 2013).

Gel/fluid transitions at physiological temperature have actually been detected in certain cell types, such as sperm cells in latent state (Wolf, 1995). In mammalian cells, a common feature of many stress responses is the rise in ceramide levels, which is likely to promote local fluid >solid transitions and/or the formation of ceramide-rich platforms (Zhang et al., 2009; Stancevic and Kolesnick, 2010).

Using atomic force microscopy to observe supported ternary lipid bilayers, Giocondi et al. (2004) could directly follow the transitions upon increasing cholesterol content by treating the bilayer with cyclodextrin loaded with cholesterol. For the 1,2-dioleoyl-*sn*-glycero-3-phosphocholine (DOPC)/bovine brain SM/cholesterol mix, increasing cholesterol resulted in the system going from gel/fluid to Lo/Ld to Lo (i.e., no domains). However, for the 1-palmitoyl-2-oleoyl-*sn*-glycero-3-phosphocholine (POPC)/bovine brain SM/cholesterol mixture, they clearly observed a gel/Lo rather than a Ld/Lo intermediate situation and suggested this to be a plausible mechanism for domain formation in biological membranes, since those contain 1-saturated-2-unsaturated phospholipids rather than 1,2-diunsaturated ones. Until recently, the coexistence of Lo phase with So in binary phospholipid/sterol mixtures had not been observed by any imaging technique. However, this situation has changed and Lo/So nanodomains have now been observed directly by high-resolution atomic force microscopy in mixtures of DPPC/ergosterol (Vanegas et al., 2010).

Direct evidence for the existence of solid domains in the PM of a eukaryote was obtained by one of us with the budding yeast *S. cerevisiae* (Aresta-Branco et al., 2011).**Using *trans*-parinaric acid, a fluorescent probe that has very different lifetimes in liquid and solid environments, it was demonstrated that, in cell cultures in mid-exponential phase, the sphingolipid-enriched domains are in a So state. Results of experiments performed with spheroplasts and a GPI-anchor remodeling mutant suggested that those domains might be points of interaction with the cell wall, possibly through GPI-anchored proteins. In addition, multiple evidence suggested that those solid domains were mostly located in the PM; they were strongly enriched in the PM fraction obtained by discontinuous gradient ultra-centrifugation, and in liposomes prepared with lipids extracted from purified PM as compared to intact cells and total lipids. A very recent report, performed by a completely independent group, has not only confirmed the formation of gel-like domains in the membranes of *S. cerevisiae*, but further documented the importance of sphingolipids in the process (Vecer et al., 2013).

At this stage, we feel that it is important to underline that the definition of a solid state in the context of biomembrane research does not equate with a frozen structure that would be completely static and rigid since such a structure would almost inevitably lead to cell death. The difficulty in using the terms of solid, liquid and gaseous is that those words are actually much better suited for describing macroscopic states. Membrane arrangements are, however, inherently micro-heterogeneous systems and highly anisotropic. Moreover, membrane microdomains are clearly micro- or even nanoscopic structures, involving only few hundreds of molecules, thus limiting the application of long-range/short-range order criteria for the distinction of phases.

Thus, when one considers things at the molecular level, time scales become a much more practical criterion to discriminate between those various states, in particular the average duration of interactions between neighboring molecules. In short, at the molecular level, one can say that in a gas molecules are separated from their neighbors most of the time, which they only encounter occasionally through collisions. In a liquid, molecules are in constant contact with their direct neighbors, but the interactions are too short for them to fully adapt their molecular structures to one another. In other words, in a liquid all intermolecular interactions last, on average, less than a microsecond. As soon as molecules start interacting with their neighbors for periods that are longer than a few microseconds, molecules will have enough time to modify their molecular architecture as a result of those interactions, and systems will start behaving more as solids than liquids (Elson et al., 2010). Under gel/solid conditions in a lipid bilayer, however, molecules still remain highly dynamic, and very significant diffusion does still occur, albeit much more slowly than in a liquid state. For example, even at 10^-2^ μm^2^/s, the typical slow lateral diffusion rate of DPPC in a gel state, it takes only about one second for any two molecules randomly distributed in a 100 nm diameter LUV to encounter one another (this was calculated using the model of Tachyia, 1987; de Almeida et al., 2002).

## THE SOLID DOCKS HYPOTHESIS

In the following paragraphs we propose a model where the formation of many of the microdomains that appear in the PM of eukaryotes involves components switching from a fluid or amorphous state to a crystal-like state, based on the specific recruitment of particular lipids and proteins into solid nanodomains.

From a thermodynamic point of view, the recruitment of lipids into a solid crystalline-like domain will be energetically favorable for those specific lipids that acquire a solid type conformation due to favorable molecular interactions with the nucleus, i.e., the solid ordered nanodomains. This “crystalline recruitment” will be specific, therefore different lipids will be recruited into different types of domains. Crystal growth is not governed solely by thermodynamics, as it requires individual molecules to diffuse and to encounter the nucleation seeds, and/or the diffusion of the nanodomains toward one another. Solid nanoclusters will grow and decay until a critical size is reached and the crystallization process continues to form a large and stable crystalline domain or platform. Hence, most of the times, the solid clusters are highly labile entities that form and collapse, which actually corresponds to features that have been attributed to lipid rafts, and not at all frozen structures as they are usually described. Furthermore, the reverse switch from a domain back to the liquid phase will tend to be much less probable, resulting in much longer times of residence inside solid than inside Lo domains. This type of specific recruitment into solid domains would thus provide a possible explanation for the parallel formation of different types of microdomains at the surface of a single cell, and over timescales that would be much more compatible with those necessary for cellular events such as intracellular transport or the assembly of signaling platforms, which usually occur over periods going from seconds to minutes or even hours (Papin et al., 2005; Hoffman et al., 2011). Incidentally, recent advances in deciphering the general process of crystallization of solutes suggest that it may often involve the initial formation of nanoparticles which subsequently join to form a larger crystal (Teng, 2013). Crystalline membrane nanodomains could thus correspond to such nanoparticles, but their growth would be limited by availability of additional components, and by cellular responses such as the constant turnover due to an intense trafficking inside the cells.

Within such a model, where crystalline structures are more stable than the fluid ones, their disruption would not simply happen by removing the stimulus that initially led to their formation. It would thus be necessary to remove the solid platform from the membrane, e.g., by endocytosis, and then follow membrane recycling processes. This is in fact known to occur for most surface receptors that form dimers or oligomers upon stimulation. Alternatively, changes in the physical environment following stimulation, such as a change in the lipid composition, in the membrane electric dipole potential, or in ionic potential could lead to a novel situation where the solid state would no longer be more stable than the fluid one. For example, it is well established that several signaling transduction pathways involve a variety of mechanisms such as activation of lipases, alteration of lipid biosynthetic rates, strong alterations in ionic gradients, and most notably in the levels of cytosolic calcium, or even the alteration of membrane binding affinity of highly cationic proteins at the cytosolic side of the PM. All these events have been recognized for some time as important control mechanisms of the fluctuations in the physical state and composition of dynamic membrane domains (Heimburg, 2003). They could in fact function as negative feedback loops, whereby a signaling platform would activate a series of cellular events which would in turn lead to the “melting” of the signaling platform.

Another important aspect of the formation of such domains concerns the homogenizing role of sterols. As was extensively developed earlier, sterols tend to act as homogenizers, by preventing the occurrence of an all-or-nothing liquid to solid transition, and by promoting the co-existence of lipids with very different *T*_m_’s into an intermediate state between Ld and So. In some cases, sterols may become incorporated into the crystalline lattice, but in most cases, given their capacity to disrupt solid phases, they would presumably have to be excluded from solid structures. Quite possibly, however, sterols could in certain instances accumulate in one leaflet to fill the gaps, for example if the crystalline lattice only involves one leaflet of the bilayer, or if that lattice involves lipids with very long fatty acid chains that can span into the opposite leaflet.

In **Figure 3**, we present an ultra-schematic representation of the dock model, where the dimerisation of a membrane receptor upon binding of its ligand provides the seed for the formation of a crystalline dock comprised of a single lipid species or lipid complex (**Figures 3A–D**). As depicted in the lower part of **Figure 3** (i.e., sequence E,F), rings would be expected to form around the crystalline docks, corresponding to an area of transition in order, thickness and other physical properties between the solid center and the more fluid general membrane environment. Such rings could even have a role to play in the enrichment of certain proteins, or of particular lipid species, for their recruitment into the central docks. In this regard, such rings would be quite akin to the lipid shells proposed by Anderson and Jacobson (2002). Such arrangements would not represent a significant entropic cost because they would have properties in between the solid platforms and the remainder of the membrane, avoiding for example a large hydrophobic mismatch. This organization of domains was actually observed in DOPC/DPPC/cholesterol GUVs (**Figure 2A**) and was also predicted, by FRET experiments, to be the one that occurs when highly ordered ceramide/sphingomyelin-enriched gel domains form within PC/SM/cholesterol membranes with Ld/Lo coexistence (Silva et al., 2007).

Earlier we alluded to recent evidence that the PM of eukaryotic cells could be mostly in a Lo phase. Since, by nature, living organisms are not in thermodynamic equilibrium, one possible way of describing the lateral organization of membranes is to consider that there are membrane regions behaving as an amorphous state. Provided that the system is near or above the so-called glass transition temperature *T*_g_, amorphous states can still show high levels of molecular diffusion (Graeser et al., 2010). Therefore, it could turn out that the Lo-like state of the PM of live cells actually corresponds to an amorphous state with high lateral diffusion.

Within the framework of an out-of-equilibrium description of the cell membrane, the density fluctuations characteristic of amorphous states could explain membrane properties that are usually ascribed to the Ld/Lo coexistence paradigm. For example, it has recently been established that, under particular thermodynamic conditions, a two-dimensional fluid can become a stable mosaic of small differently ordered clusters which can be described as a state of micro-phase separation between amorphous and crystalline domains (Patashinski et al., 2013).

An amorphous state is not an equilibrium state and is thus of high Gibbs energy (aka free energy or free enthalpy). Consequently, it may present different degrees of short range order and rigidity. The formation of the crystalline domains would entail the use of that high energy, via a transition from amorphous to crystalline state. The discussion in the previous paragraphs about nucleation and microdomain formation does, however, remain valid because the mechanism for crystal growth is the same as for a liquid-to-crystalline transition (Hancock and Zografi, 1997). Furthermore, another characteristic of the amorphous state is the presence of density fluctuations (Wiegand, 1979), and composition fluctuations assigned to critical behavior were observed in membrane model systems of lipid rafts (i.e., with liquid-liquid phase separation) and also in giant PM vesicles (Honerkamp-Smith et al., 2009).

As underlined by Quinn (2010), micro-phase separation can also be observed in model bilayers comprised of ternary mixtures of asymmetric sphingolipids, glycerolipids and cholesterol. Their examination by a number of biophysical methods has led to a set of results that could be interpreted as formation of binary complexes of asymmetric sphingolipids and glycerophospholipids that exclude cholesterol, and which have diffusional characteristics more akin to a gel than a Lo phase (Filippov et al., 2006). In the past, the group of Quinn has characterized quasi-crystalline phases in co-dispersions of phosphatidylethanolamine and glucocerebroside (Feng et al., 2004). More recently, Quinn (2010) has proposed a “lipid matrix model” for the formation of rafts, whereby the seeding of stoichiometric complexes between certain sphingolipid and phospholipid species with a highly ordered or quasi-crystalline organization would result in solid-like phases in biological membranes under physiological conditions. According to this model, asymmetric sphingolipids with very long chain fatty acids (e.g., C24), even if only present in small amounts in cell membranes, are responsible for the formation of quasi-crystalline phases which exclude cholesterol; the glycosyl component of those sphingolipids would provide additional selectivity through interaction with proteins. This type of model for the co-existence of different types of solid domains is supported by extensive experimental data obtained with model membranes made of binary lipid mixtures which showed solid-solid immiscibility even when the lipids differed only in their headgroup or in the acyl chain length (Mabrey and Sturtevant, 1976; Silvius and Gagne, 1984; Graham et al., 1985; Marsh, 2013).

*In fine*, the “Lipid matrix model” proposed by Quinn (2010) is effectively very similar to the model of solid docks (Joly, 2004). In both cases, the basic idea is that different solid-phase seeds would exist, mostly due to proteins inducing different crystalline lattices, thus leading to the formation of different domains that would specifically recruit certain solid-forming lipids. Since such lipids only comprise a small percentage of the membrane lipids, the growth of those domains would be self-limiting, but would be expected to suffice for the recruitment of additional proteins, either identical to or different from those having seeded the dock. Good candidates for proteins that could promote and/or stabilize such domains are surface receptors that tend to homo- or hetero-dimerize (or even multimerize) upon stimulation (as stated above, stable crystal growth depends on the attainment of a minimum critical size of the nucleation center), as well as components anchored to cell-wall proteins in fungi and plants, and cytoskeleton proteins in all eukaryotes. Such components would have a major influence on the movements of all the dock’s components, and consequently also on the diffusion of the free molecules in the direct vicinity of the dock, which would explain why disrupting the cytoskeleton can have such a dramatic effect on the existence of PM microdomains and/or on the diffusion of proteins and lipids that do not directly interact with cytoskeleton components.

## TECHNICAL CHALLENGES

To date, only a handful of studies have documented, or even just suggested the existence of solid domains in eukaryotic membranes. This could be taken to imply that such solid domains only occur exceptionally, but we suspect that this may actually be due more to the fact that people have not looked for them, which is in large part related to the almost complete absence of clear-cut and/or foolproof methods for the detection of such solid nanodomains in living cells under physiological conditions. Furthermore, and as underlined earlier, if one considers the amount of sterols in the PM of all eukaryotic cells, comparison with lipid phase diagrams established in membrane model systems leads to the prediction that most of the PM will be in a Lo state, in agreement with recent results obtained in cells. Other types of domains would thus be a minority, and therefore difficult to detect. We do, however, remain convinced, and hopeful, that people will start to find evidence for the existence of solid domains in the PM of eukaryotic cells when they start looking for them, with the proper tools. In this respect, Heimburg (2003) has pointed out that the absence of a pronounced melting peak in many biological systems cannot be directly interpreted as an absence of melting events, since the chain melting may be spread out over a large temperature range due to the large variety of lipid components, rendering it difficult to detect such transitions.

One of the main hurdles for the characterization of microdomains in live cells has been attributed to their small size of a few tenths of nanometers, and thus below the diffraction limit of visible light, as well as their very dynamic nature, which have prevented their direct observation by standard light microscopy. Over the past few years, a score of technological developments have, however, been pushing back the limits of this resolution well below 100 nm, and a review focused on the advances of super-resolution microscopy relevant to understanding membrane lipid domains can be found in the same issue as our paper (Owen and Gaus, 2013). For example, using STED-FCS to reach resolutions of the order of 30 nm, Mueller et al. (2011) documented anomalous diffusion of sphingolipid probes at the surface of live cells, leading them to suggest the existence of domains involving strong intermolecular interactions either between lipids, or between lipids and proteins.

To date, a very large proportion of studies that have been carried out on live cells have relied on approaches that were not suited to discriminate between Lo and So phases. For example, fluorescent probes such as Laurdan or di-4-ANEPPDHQ have been extensively used to study membrane order, but those probes do not allow to fully discriminate between either Lo and So or So and Ld phases. To document the existence of solid domains in a variety of cells, comparison of anisotropy values with probes such as DPH could be a more hopeful approach, but remains limited in its capacity to detect nanoscopic solid domains, among other disadvantages such as UV excitation wavelengths or photoinstability. Approaches based on the fluorescence lifetimes of certain fluorescence probes can lead to the definitive detection of solid phases, as was achieved in yeast with tPNA (Aresta-Branco et al., 2011; Vecer et al., 2013). The very high sensitivity of this probe to photo-degradation does, however, preclude its use for microscopy. None of the above probes are thus ideal to discriminate between So and Lo domains in single cells, and new probes that are photostable and easy to incorporate into cells are still sorely needed. In this regard, the combination of molecular rotor probes and FLIM imaging seems a very hopeful approach since it was used to document that the microviscosity of the inner membrane of *B. subtilis* dormant spores was very high, suggesting that it was in a gel state. Interestingly, this viscosity decreased significantly upon germination (Loison et al., 2013). One of the main difficulties in using fluorescent probes to investigate the existence of crystalline domains, however, is that, if there is a specificity of recruitment into crystalline nanodomains, many probes will most likely be excluded from such domains.

NMR spectroscopy provides a definite way to discriminate between Lo and So states, but the studies that have used FTIR and NMR spectra to study membrane order in mammalian cells have almost exclusively relied on the incorporation of deuterated cholesterol derivatives, which one would expect to be excluded from solid domains. Notwithstanding the fact that solid nanodomains are only ever expected to represent a minute portion of a cell’s PM, and that NMR-based studies do not distinguish into which lipid species the isotopic labeled component was integrated, it would still be interesting to carry out similar experiments with deuterated lipid precursor(s) that would incorporate preferentially into solid-forming lipids such as sphingolipids with long chain fatty acids.

Another approach that may be used to assess whether or not solid lipid phases are present in signaling platforms may be derived from that described by Harder and Kuhn (2000) in which they incubated lymphocytes with magnetic beads coated with anti-T-cell receptor antibodies and fragmented the membranes by cavitation before isolating the membranes containing the signaling complexes surviving the mechanical disruption process. As an alternative, we have also used exosomes as a source of naturally isolated microdomains, and found them to be in a very ordered state by DPH anisotropy measurements (Carayon et al., 2011), as well as Laurdan spectroscopy and tPNA lifetime measurements (unpublished data). Chemical mapping after metabolic labeling, such as developed by the group of Mary Kraft is yet another hopeful approach (Kraft, 2013), although this only gives information on the concentration enrichment of a given lipid species, but does not indicate which kind of phase the lipids are in.

With the tools and methodological approaches available today, the study of membrane lipid domains in physiological conditions thus remains a difficult problem to tackle and the design of clear-cut experiments that will allow the unquestionable discrimination between Lo and So phases will first require that scientists start looking for solid domains, but will also probably have to await the development of new tools, and/or new approaches.

## CONCLUSION

From the perspective of the fluid mosaic model, the formation of gel/So/crystalline domains in biological membranes was initially perceived by many as very unlikely, but experimental evidence keeps accumulating suggesting their possible existence. Furthermore, the formation of such crystalline nanodomains could confer the stability required for the kind of time frames and selectivity for cellular events to unfold, such as intercellular transport or assembly of signaling platforms.

## AUTHOR CONTRIBUTIONS

Rodrigo F. M. de Almeida and Etienne Joly contributed equally to the ideas developed in this manuscript, and to its writing.

## Conflict of Interest Statement

The authors declare that the research was conducted in the absence of any commercial or financial relationships that could be construed as a potential conflict of interest.
